# Isolation and Characterization of Novel Murine Epiphysis Derived Mesenchymal Stem Cells

**DOI:** 10.1371/journal.pone.0036085

**Published:** 2012-04-27

**Authors:** Chun-Chun Cheng, Wei-Shiung Lian, Felix Shih-Hsiang Hsiao, I-Hsuan Liu, Shau-Ping Lin, Yen-Hua Lee, Chia-Chun Chang, Guan-Yu Xiao, Hsin-Yi Huang, Ching-Feng Cheng, Winston Teng-Kuei Cheng, Shinn-Chih Wu

**Affiliations:** 1 Institute of Biotechnology, National Taiwan University, Taipei, Taiwan; 2 Department of Medical Research, Tzu Chi General Hospital and Department of Pediatrics, Tzu Chi University, Hualien, Taiwan; 3 Institute of Biomedical Sciences, Academia Sinica, Taipei, Taiwan; 4 Department of Animal Science and Technology, National Taiwan University, Taipei, Taiwan; 5 Research Center for Developmental Biology and Regenerative Medicine, National Taiwan University, Taipei, Taiwan; 6 Department of Animal Science and Biotechnology, Tunghai University, Taichung, Taiwan; University of Torino, Italy

## Abstract

**Background:**

While bone marrow (BM) is a rich source of mesenchymal stem cells (MSCs), previous studies have shown that MSCs derived from mouse BM (BMMSCs) were difficult to manipulate as compared to MSCs derived from other species. The objective of this study was to find an alternative murine MSCs source that could provide sufficient MSCs.

**Methodology/Principal Findings:**

In this study, we described a novel type of MSCs that migrates directly from the mouse epiphysis in culture. Epiphysis-derived MSCs (EMSCs) could be extensively expanded in plastic adherent culture, and they had a greater ability for clonogenic formation and cell proliferation than BMMSCs. Under specific induction conditions, EMSCs demonstrated multipotency through their ability to differentiate into adipocytes, osteocytes and chondrocytes. Immunophenotypic analysis demonstrated that EMSCs were positive for CD29, CD44, CD73, CD105, CD166, Sca-1 and SSEA-4, while negative for CD11b, CD31, CD34 and CD45. Notably, EMSCs did not express major histocompatibility complex class I (MHC I) or MHC II under our culture system. EMSCs also successfully suppressed the proliferation of splenocytes triggered by concanavalin A (Con A) or allogeneic splenocytes, and decreased the expression of IL-1, IL-6 and TNF-α in Con A-stimulated splenocytes suggesting their anti-inflammatory properties. Moreover, EMSCs enhanced fracture repair, ameliorated necrosis in ischemic skin flap, and improved blood perfusion in hindlimb ischemia in the *in vivo* experiments.

**Conclusions/Significances:**

These results indicate that EMSCs, a new type of MSCs established by our simple isolation method, are a preferable alternative for mice MSCs due to their better growth and differentiation potentialities.

## Introduction

Friedenstein and colleagues first defined mesenchymal stem cells (MSCs) in the 1970s as cells that are capable of self-renewal and possess multipotency [1]. Over decades MSCs have been shown to not only be able to differentiate into three mesodermal lineages, including adipocytes, osteocytes and chondrocytes [2], [3], [4], but also into cells types with non-mesenchymal lineages, such as hepatocytes [5], [6], pancreatic-like cells [7], [8], [9] and neuron-like cells [10], [11]. Hence, MSCs have become an attractive cell source for use in regenerative medicine. In addition, the low immunogenicity of MSCs makes them suitable for use in transplantation [12], [13], [14], and their immunomodulatory properties make them suitable for use in the treatment of many immune disorders [15], [16], [17].

MSCs were initially obtained from bone marrow [1], [4], but they can also be derived from other sources, such as skeletal muscle [18], umbilical cord blood [19], [20], dental pulp [21], adipose tissue [22], [23] and amniotic fluid [24], [25]. MSCs have been successfully isolated and expanded from human [4], rat [26], rabbit [27], canine [26], pig [28] and mouse [29]. Mouse is the most widely used species in laboratory research because they are easy to manipulate and their genetic information is readily available. However, murine is the most difficult species to establish MSCs from BM [30]. Murine BM is composed of heterogeneous cell populations that contain few MSCs (10^−5^–10^−6^ cells) [31]. In addition, BMMSCs are located near the inner surface of the bone, making it difficult to flush them out [32]. Another problem in establishing mouse BMMSCs is contamination with large amount of hematopoietic cells [33]. Therefore, it is necessary to expand MSCs *ex vivo*. Such manipulation could cause cellular senescence by the loss of proliferation, differentiation and therapeutic potentials [34], [35]. This prompted us to look for an alternative source for MSCs with better *ex vivo* expansion capability.

Endochondral ossification occurs during the process of long bone formation in foetal development. Primary ossification occurs at the bone centre for forming marrow cavity, while secondary ossification is formed in the bone epiphysis, followed by the formation of uncalcified cartilage, perichondrium and epiphyseal blood vessel penetration [36], [37], [38]. Hence, we hypothesized the possibility of a biological niche for mesenchymal progenitors in the epiphysis. In this study, we derived novel MSCs from murine epiphysis without enzymatic digestion. We characterized the morphology, proliferation and functional properties of EMSCs and compared these results with those of BMMSCs under the same cell culture conditions. We also evaluated the therapeutic effects of EMSCs on bone fracture and two types of ischemia mouse animal models. To our knowledge, this is a novel approach for the isolation of MSCs from murine bone.

## Results

### Establishment of EMSCs

**Figure 1:**
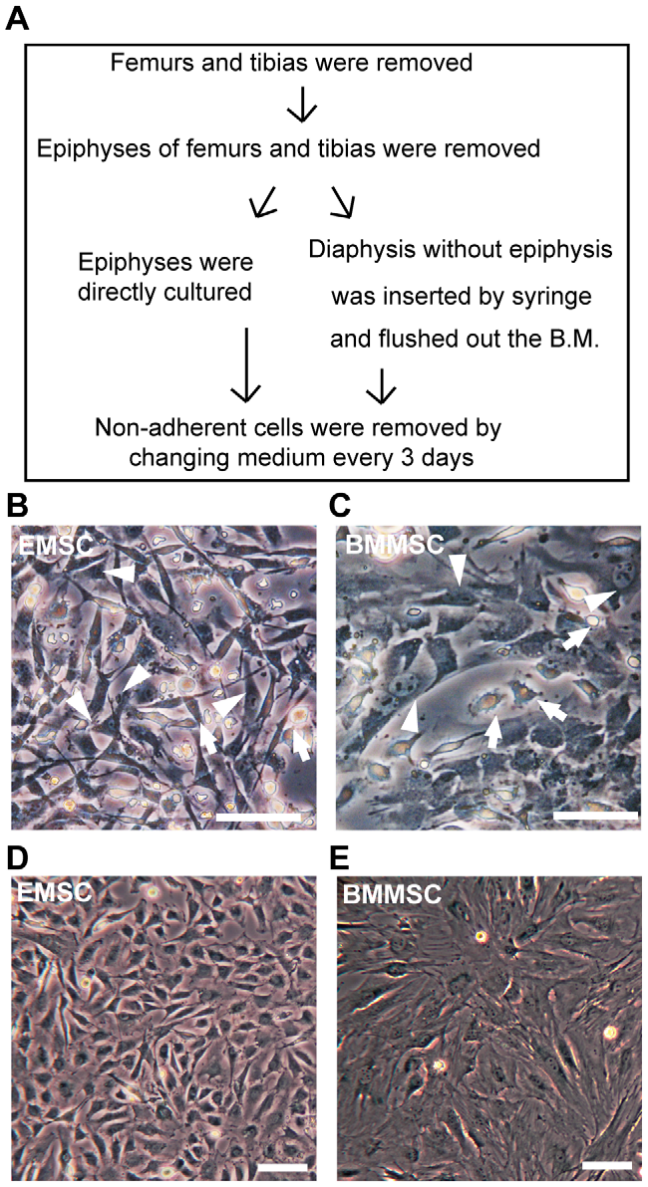
Establishment of EMSCs and BMMSCs. (A) Schematic protocol for the establishment of cultures of EMSCs and BMMSCs. (B) Phase-contrast micrograph of EMSCs in primary culture after seven days. Arrow heads indicate triangle, spindle-shaped, fibroblast-like EMSCs. Lymphohematopoietic cells are indicated by arrows. (C) Phase-contrast micrograph of BMMSCs in primary culture after seven days. Arrow heads indicate flat, fibroblast-like BMMSCs and arrows indicate lymphohematopoietic cells. (D) Phase contrast micrograph of EMSCs at the fifth passage showing that EMSCs maintained their shape during propagating. (E) Phase contrast micrograph of BMMSCs upon the fifth passage showing that BMMSCs became flatted with increasing passages. All the scale bars represent 100 µm.

Because surface antigens specific to MSCs have not been identified, MSCs are mainly isolated using their characteristic of plastic adherence. We obtained BMMSCs using a BM flush-out method and EMSCs using our newly developed method for acquiring MSCs (Figure 1A). Epiphysis was dissected out and directly cultured in culture dishes without enzymatic digestion. After seven days of culturing, EMSCs can be observed as triangle, spindle-shaped (Figure 1B), while BMMSCs had a flat, spindle-shaped morphology (Figure 1C). Since both methods had the same problem of hematopoietic cells contamination, we purified the primary culture of EMSCs migrating from epiphysis and BMMSCs from bone marrow by the transient lower-density plastic adherence (tLDA) method [39] to avoid hematopoietic cells contamination. In subsequent passages, EMSCs maintained their characteristic spindle-shape (Figure 1D), whereas BMMSCs changed their morphology and displayed enlarged and flattened phenotypic appearances (Figure 1E).

### Characterization of EMSCs

**Figure 2:**
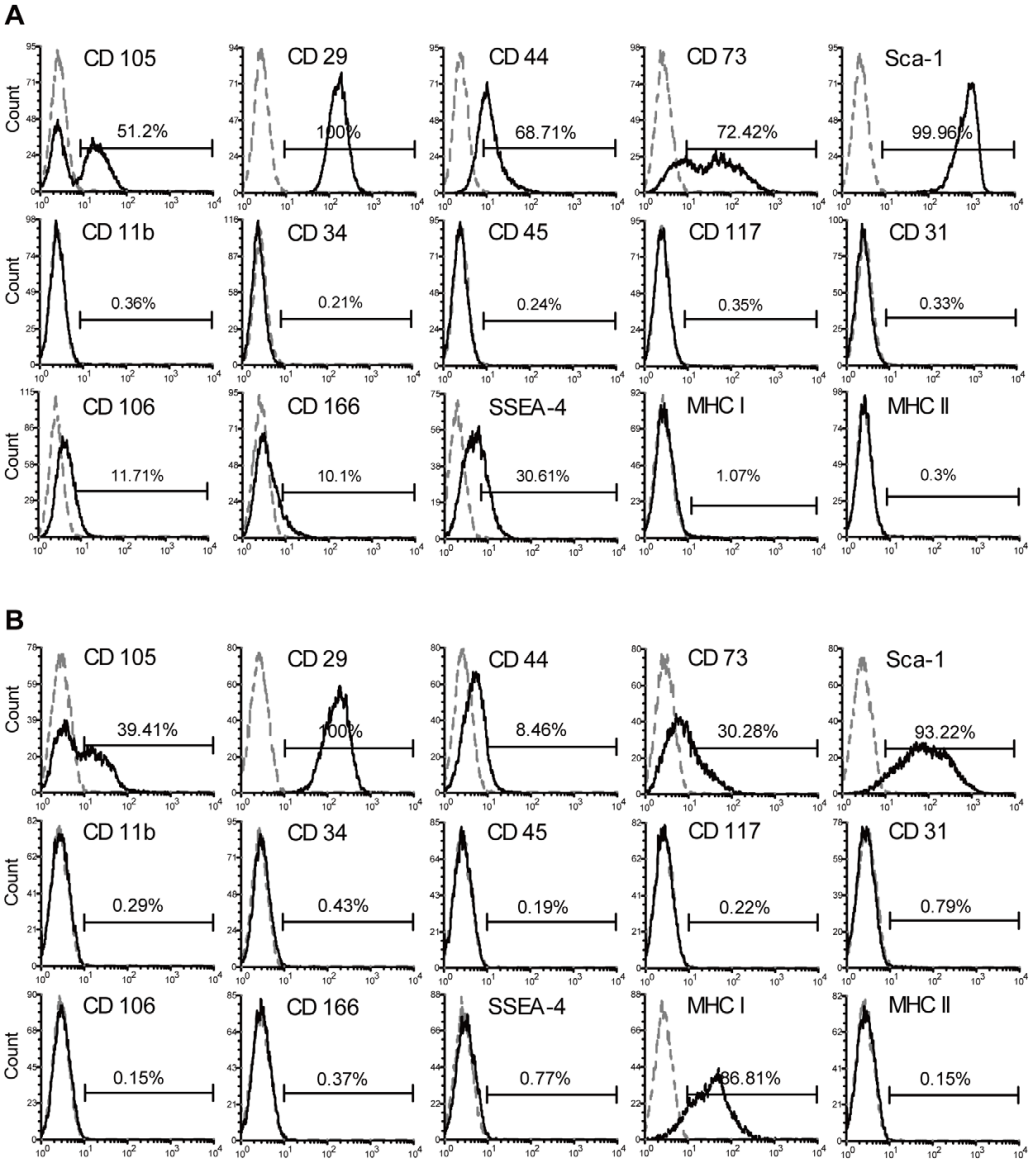
Immunophenotypes of EMSCs and BMMSCs. (A) Flow cytometric analysis of markers related to stem cells, mesenchymal stem cells, hematopoietic cells, endothelial cells and immune cells on EMSCs and (B) BMMSCs at the first passage. The dotted line indicates the respective isotype control.

To verify the cells derived from the epiphysis are MSCs, a panel of antibodies against stem cells markers was chosen to evaluate whether the phenotypes of EMSCs are similar to MSCs. EMSCs were positive for most positive BMMSCs markers, such as CD29, CD44, CD73, CD105 (Figure 2A) and with stronger Sca-1 signal than BMMSCs (Figure 2A, 2B), while negative for hematopoietic cells markers, CD11b, CD45 and CD34, and for endothelial cell marker, CD31 (Figure 2A) as BMMSCs (Figure 2B). In addition, EMSCs were positive for markers, CD106, CD166 and SSEA-4 (Figure 2A), while BMMSCs were negative (Figure 2B).

**Figure 3:**
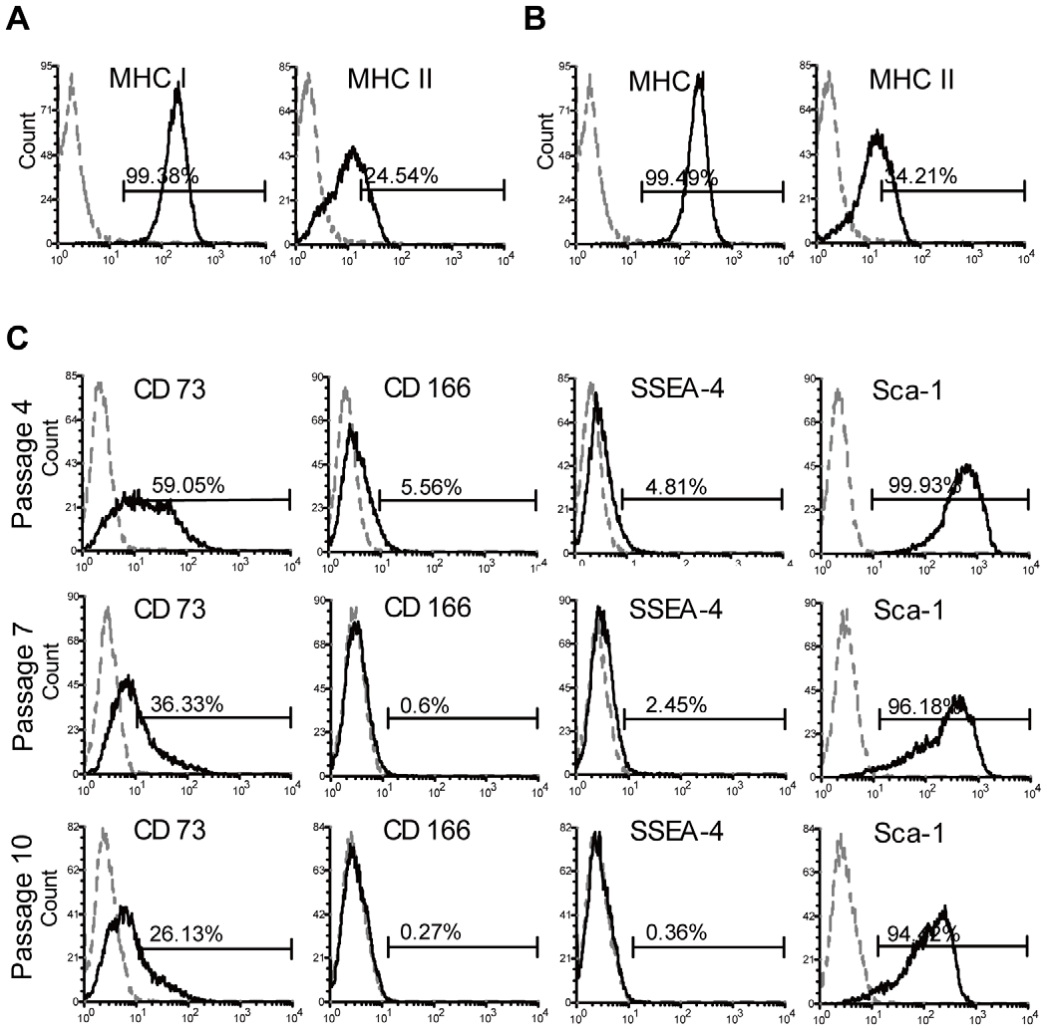
Surface markers analysis of EMSCs after IFN-γ treatment and during increased serial passages. (A) MHC I and MHC II expression profiles for EMSCs (passage five) were analysed under 10 ng or (B) 100 ng IFN-γ treatment. EMSCs were positive for MHC I and MHC II after IFN-γ treatment. (C) EMSCs surface antigen profiles were detected via flow cytometric analysis during increasing passages. The markers of CD73, CD166, SSEA-4 and Sca-1 were decreased with propagating. Respective isotype control is indicated by the dotted line.

MSCs have reportedly low immunogenicity, because MHC II is not expressed. We found that EMSCs were also negative for MHC II (Figure 2A). Interestingly, EMSCs were negative for MHC I (Figure 2A), whereas BMMSCs were positive (Figure 2B). To test whether the EMSCs are able to express MHC I, we treated EMSCs with 10 ng and 200 ng IFN-γ (Figure 3A, 3B, respectively) and detected the MHC molecules expressions. Although MHC class I and II molecules were not expressed by EMSCs under our isolation method and culture conditions, their expression could be induced by IFN-γ treatment.

Furthermore, we monitored immunophenotype changes that occurred during serial passages. Among the antibody panel used, four surface markers gradually decreased with increased serial passage, including CD73, CD166, SSEA-4 and Sca-1 (Figure 3C), which became more similar to BMMSCs (Figure 2B) but the MHC I expression was still negative even with additional passages (data not shown).

### EMSCs Have Better Proliferation Potential

**Figure 4:**
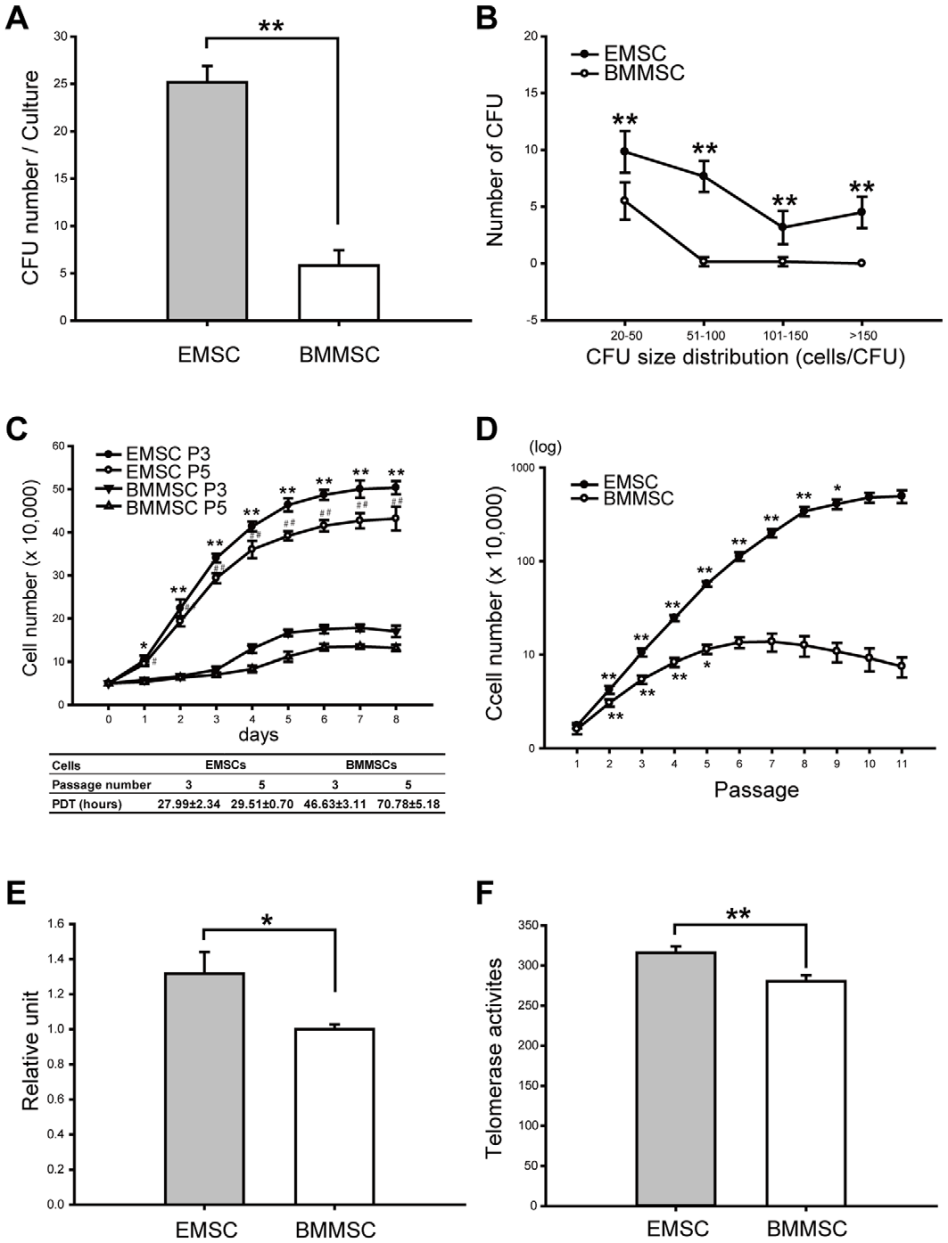
Cell proliferation profiles of EMSCs and BMMSCs. (A) Colony-forming activity of EMSCs and BMMSCs at the first passage. EMSCs formed 23.17±2.92 CFUs whereas BMMSCs formed 5.83±1.60 CFUs (n = 6). (B) CFUs size distribution for EMSCs and BMMSCs. EMSCs formed more and larger CFUs than BMMSCs. ** represents *P*<0.01 (n = 6). (C) PDTs for EMSCs were compared to BMMSCs at the third (P3) and the fifth (P5) passages. The curves of EMSCs are higher than the ones of BMMSCs (n = 3). * represents *P*<0.05, ** represents *P*<0.01, which was compared at the third passage. # represents *P*<0.05, ## represents *P*<0.01, which was compared at the fifth passage. (D) Propagating abilities of EMSCs and BMMSCs. Cells were propagated for 2 days at each passage and cell numbers were calculated. EMSCs could be propagated for more passages that cells still retained proliferation ability than BMMSCs (n = 5). * represents *P*<0.05, ** represents *P*<0.01, as compared to previous passage. (E) Telomere length and (F) telomerase activity of EMSCs and BMMSCs (at the first passage) were detected and compared. EMSCs expressed significantly longer telomere length and higher telomerase activity than BMMSCs (n = 3). * represents *P*<0.05, ** represents *P*<0.01. Data are presented as mean ± s.d. and analyzed with Student's *t*-test.

We evaluated EMSCs proliferation potential using a colony-forming assay. EMSCs formed 23.17±2.92 CFUs, showing significantly higher clonogenic ability than BMMSCs (5.83±1.60 CFUs) (Figure 4A). EMSCs also contained more CFUs that grow into large colonies than BMMSCs (Figure 4B).

The population doubling time (PDT) assay was performed to evaluate the proliferation ability of EMSCs. The curves of both EMSCs at the third and the fifth passages were higher than BMMSCs (Figure 4C), and the PDTs for EMSCs (27.99±2.34 hours at the third passage and 29.51±0.70 hours at the fifth passage) were less than that for BMMSCs (46.63±3.11 and 70.78±5.18 hours) (Figure 4C). Hence, EMSCs showed higher proliferation capability than BMMSCs. We further investigated the propagating ability of EMSCs, and the data showed that EMSCs could be significantly propagated until passage nine in which the cell number was not significantly increased in next passage (Figure 4D). In contrast, BMMSCs could only be propagated until passage five (Figure 4D). Thus, EMSCs can reach to a higher propagating passage than BMMSCs and have higher proliferative potential.

Since telomere length and telomerase activity are in relation to cell proliferation and aging, we further evaluated these properties in EMSCs and BMMSCs, and according by both telomere length and telomerase activity were greater in EMSCs than in BMMSCs (Figure 4E, 4F). Hence, EMSCs derived by our protocol showed higher proliferation potential.

### EMSCs Show Higher Multipotent Potentials than BMMSCs

**Figure 5:**
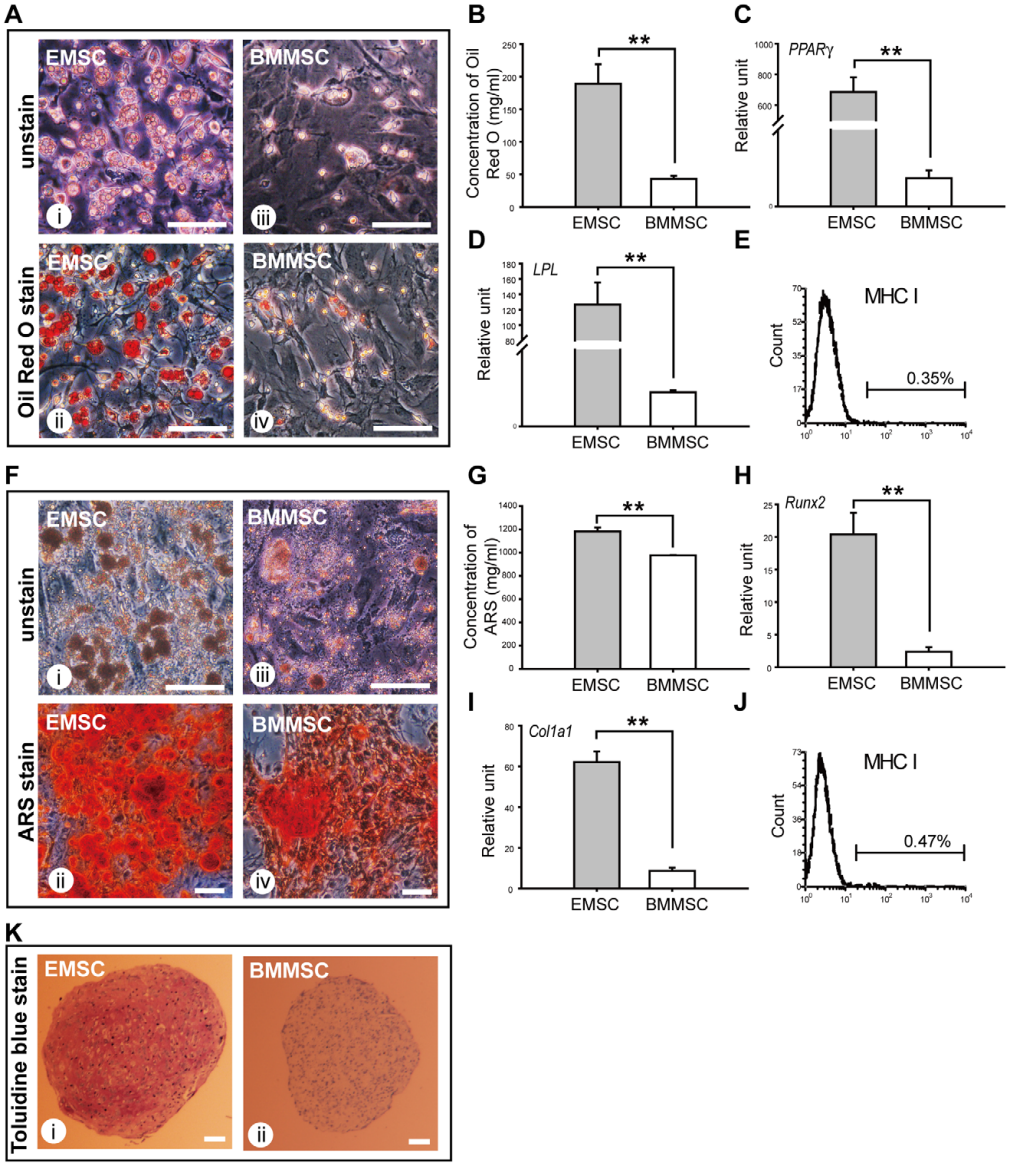
Differentiation potential of EMSCs and BMMSCs. (A) Adipogenic differentiation of EMSCs and BMMSCs at the fifth passage. Adipogenic capability was characterized by Oil Red O staining after seven days induction. EMSCs showed larger and more lipid drops than BMMSCs. (B) Oil Red O staining of EMSCs and BMMSCs were quantified for comparison (n = 3). (C, D) Expression level of adipogenic related genes were assessed by qPCR. EMSCs showed higher adipogenic gene expression level after seven days induction (n = 3). (E) MHC I expression profile of EMSCs was analysed by flow cytometry after seven days adipogenic induction. After adipogenesis, EMSCs were still negative for MHC I expression. (F) Osteogenic differentiation of EMSCs and BMMSCs at the fifth passage. Osteogenic capability was characterized by ARS staining after seven days induction. EMSCs showed higher calcium deposition than BMMSCs. (G) Quantitative results of ARS were performed to compare the osteogenic capacity of EMSCs and BMMSCs (n = 3). (H, I) Expression level of osteogenic related genes were assessed by qPCR. EMSCs showed higher osteogenic gene expression level after seven days induction (n = 3). (J) MHC I expression profile of EMSCs was analysed by flow cytometry after seven days osteogenic induction. After osteogenesis, EMSCs were still negative for MHC I expression. (K) Chondrogenic differentiation of EMSCs and BMMSCs at the fifth passage. Chondrogenic capability was evaluated by histological section of micromass pellet cultures after 21 days induction. Glycosaminoglycan content of the pellet was characterized by toluidine blue staining. EMSCs showed deeper purple stained matrix than BMMSCs. All the scale bars represent 100 µm. ** represents *P*<0.01. Data are presented as mean ± s.d. and analyzed with Student's *t*-test.

Previous studies have demonstrated that MSCs are capable of differentiation into osteocytes, adipocytes and chondrocytes. Firstly, we investigated and compared the *in vitro* adipogenic differentiation capability by culturing EMSCs and BMMSCs in adipogenic induction medium and performing histochemical staining with Oil Red O. EMSCs formed more intracellular lipid droplets upon staining (Figure 5A-i, 5A-ii) compared to BMMSCs (Figure 5A-iii, 5A-iv). Lipid droplets observed in EMSCs were also larger and more apparent. Quantitative analysis of Oil Red O staining showed that EMSCs had significantly better adipogenic differentiation capability than BMMSCs (Figure 5B). Accordingly, quantification of the adipogenic gene expression also showed that EMSCs had stronger expression both in early adipogenesis marker PPARγ (Figure 5C) and late adipogenesis marker LPL (Figure 5D). Furthermore, EMSCs maintained negative expression of MHC I during adipogenesis (Figure 5E).

Secondly, after a seven-day culture period in osteogenic induction medium, EMSCs formed calcium deposition and mature nodules structures, which were stained with Alizarin red S (ARS) (Figure 5F-i, 5F-ii). Compared to BMMSCs (Figure 5F-iii, 5F-iv), EMSCs formed relatively more nodules. Quantitative results from ARS staining showed that EMSCs had better osteogenic differentiation capability than BMMSCs (Figure 5G). The qPCR data showed greater expression of early osteogenic marker Runx2 (Figure 5H) and late osteogenic marker Colla1 (Figure 5I) in EMSCs than in BMMSCs. Furthermore, EMSCs maintained negative expression of MHC I during osteogenesis (Figure 5J).

Finally, the chondrogenic differentiation capability of EMSCs and BMMSCs were analyzed using a pelleted micromass culture system. After 21 days of chondrogenic induction culture, EMSCs differentiated into chondrocytes. The formation of chondrocytes was confirmed with toluidine blue staining, shown as a purple-colour stained matrix (Figure 5K-i). EMSCs showed more toluidine blue positive staining, whereas BMMSCs were only faintly stained (Figure 5K-ii) suggesting that EMSCs were more efficient in chondrogenesis.

Although both EMSCs and BMMSCs were able to differentiate into mesodermal cell lineages including adipocytes, osteocytes and chondrocytes, EMSCs showed higher potential to differentiate into these lineages.

### Senescence of EMSCs during Culture

**Figure 6:**
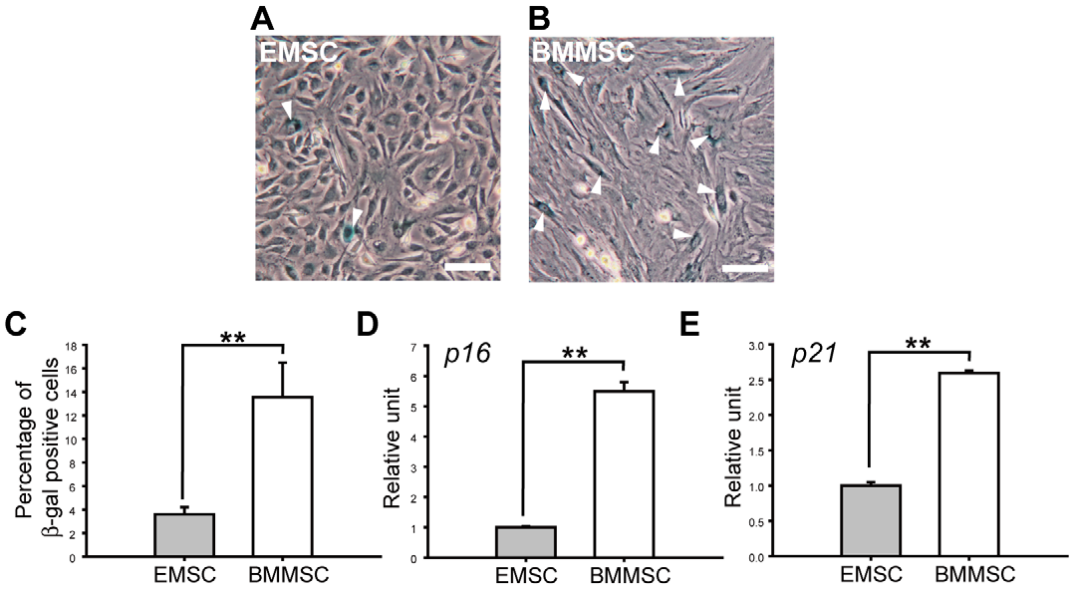
Analysis of cellular aging related markers of EMSCs and BMMSCs. (A) Determination of senescence marker, SA-β-gal, upon the fifth passage of EMSCs and (B) BMMSCs. SA-β-gal positive cells were showed as blue color and indicated by arrow heads. (C) The number of SA-β-gal positive cells was calculated from EMSCs or BMMSCs cultures. EMSCs showed lower SA-β-gal positive cells than BMMSCs (n = 4). (D) mRNA levels of cyclin-dependent kinase inhibitors p16 and (E) p21 were evaluated by qPCR. Both p16 and p21 expression levels were lower in EMSCs than BMMSCs (n = 3). All the scale bars represent 100 µm. ** represents *P*<0.01. Data are presented as mean ± s.d. and analyzed with Student's *t*-test.

Senescence can be identified not only by morphological evaluation but also by the expression of senescence-associated β-galactosidase (SA-β-gal). Blue SA-β-gal-positive cells could be found in both EMSCs (Figure 6A) and BMMSCs cultures (Figure 6B). Quantified data showed that there were more SA-β-gal-positive cells in BMMSCs than in EMSCs (Figure 6C).

We further examined the cell cycle regulators (cyclin-dependent kinase inhibitors) and senescence markers, p16 and p21. The transcript expression level of both *p16* and *p21* (Figure 6D, 6E) were lower in EMSCs than in BMMSCs.

### EMSCs are Immunosuppressive and Anti-inflammatory

**Figure 7:**
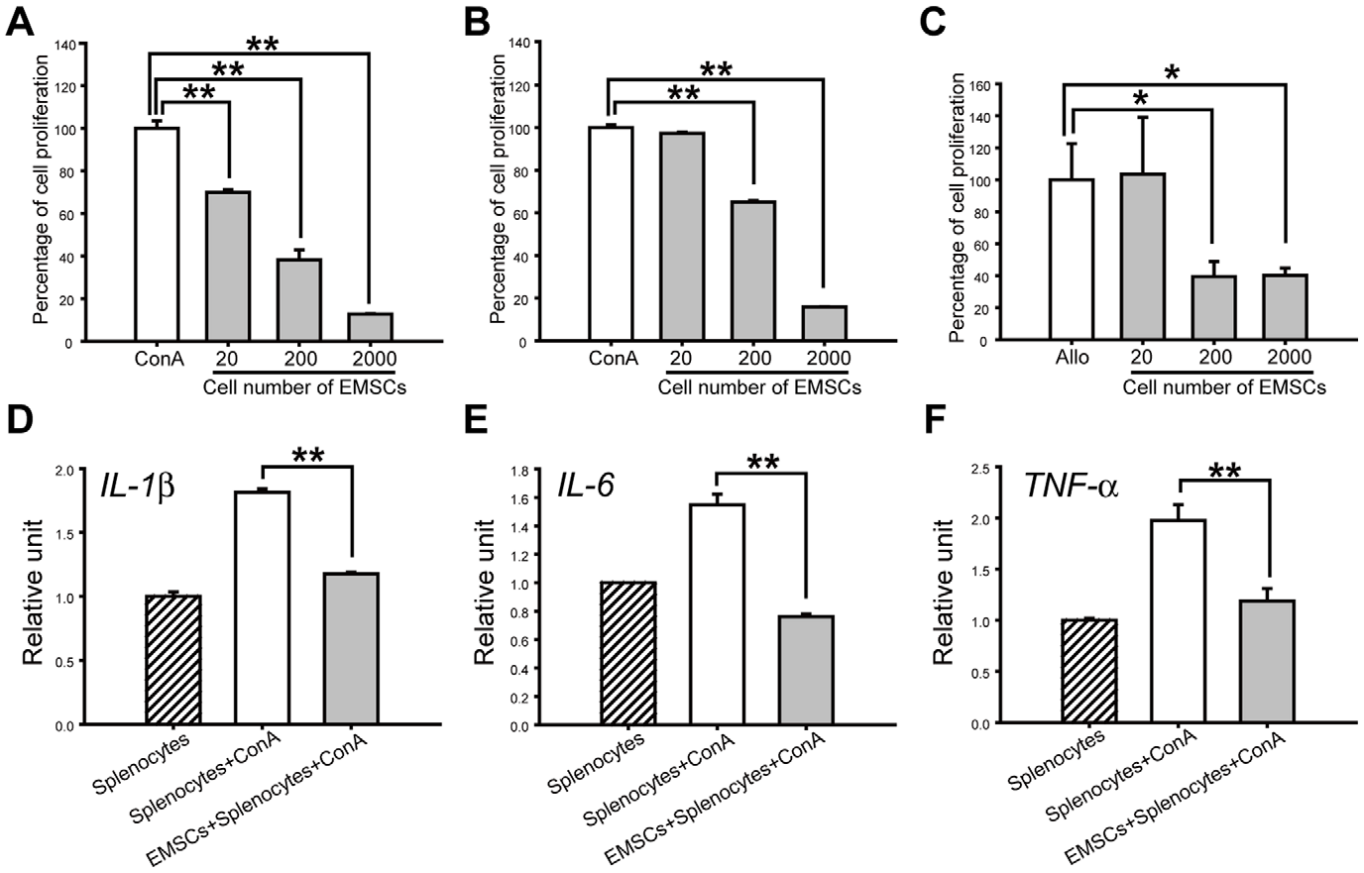
Immunomodulatory properties of EMSCs. (A) Responding splenocytes from C57BL/6 or (B) BALB/c mice were stimulated with Con A in the presence or absence of graded numbers of C57BL/6 EMSCs. The results are shown as percentage of cell proliferation in comparison with control cell proliferation. Splenocytes proliferation was inhibited by culture with EMSCs (at the fifth passage) in a dose dependent manner (n = 3). (C) Responding splenocytes from C57BL/6 mice were cultured with an equal number of mitomycin C-treated BALB/c splenocytes and with or without graded number of C57BL/6 EMSCs (n = 3). Allo indicates allogeneic splenocytes culture. EMSCs inhibited the splenocyte proliferation stimulated by allogeneic cells in a dose dependent manner. (D–F) Analysis of inflammatory cytokine production of splenocyte stimulated by Con A. Inflammatory cytokines expression level of splenocytes were decreased when co-cultured with EMSCs (at the fifth passage) (n = 3). * represents *P*<0.05, **represents *P*<0.01. Data are presented as mean ± s.d. and analyzed with Student's *t*-test.

MSCs show low immunogenicity and exhibit the ability to suppress T-cell proliferation stimulated by either non-specific lymphocyte mitogens or mix lymphocyte reaction. We speculated that EMSCs have similar features. EMSCs co-cultured with autologous C57BL/6 splenocytes in the presence of Con A significantly inhibited Con A-induced splenocyte proliferation in a dose-dependent manner (Figure 7A). For EMSCs co-cultured with allologous BALB/c splenocytes stimulated by Con A, the same effects could be observed (Figure 7B). In addition, EMSCs also repressed the allogeneic-induced splenocyte proliferation when co-cultured with allogeneic-stimulated splenocytes. Similarly, EMSCs exhibited dose-dependent suppression with allogeneic T-cell response (Figure 7C). These results indicated that EMSCs were able to suppress splenocyte proliferation *in vitro* and suggested EMSCs' role in immunosuppression.

To determine if anti-inflammatory actions of EMSCs could be induced through a paracrine effect, EMSCs and splenocytes were separated using the trans-well culture system, and IL-1, IL-6 and TNF-α of splenocytes were quantified. We found that IL-1, IL-6 and TNF-α were enhanced when splenocytes were stimulated by Con A and the surge of these pro-inflammatory signals could be repressed with the presence of EMSCs (Figure 7D–F). Hence, EMSCs are immunosuppressive via suppressing T-cell proliferation and inhibiting the release of pro-inflammatory signals.

### Therapeutic Potentials of EMSCs

**Figure 8:**
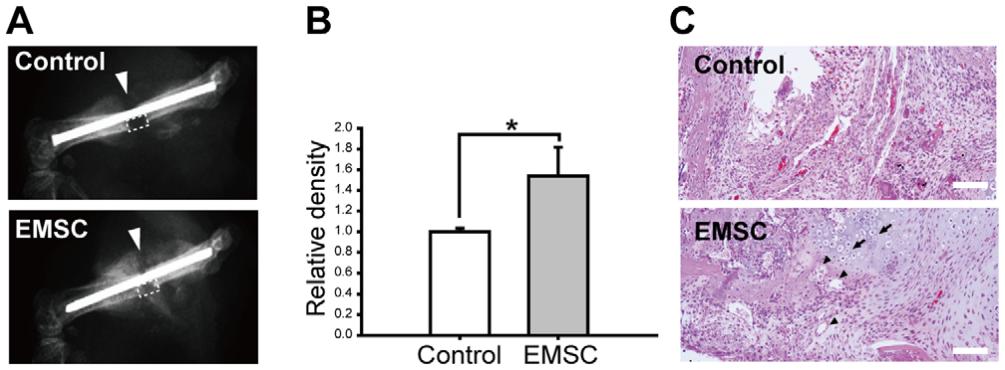
Repair of bone fracture in mice after transplantation of EMSCs. (A) Fracture healing was assessed by X-ray after 14 days with or without EMSCs (at the fifth passage) transplantation. Arrowheads indicate the site of fracture. (B) The bone density of fracture site (dotted box in Figure 8A) was quantified (n = 4). EMSCs transplantation significantly improved osteocalcification as compared to the control group. (C) The morphology of fracture sites were examined by H&E staining. Tissue disorganization was showed in the control group while the EMSCs transplanted group newly formed bone tissue (arrow) and capillaries (arrowhead). * represents *P*<0.05. Scale bars represent 100 µm. Data are presented as mean ± s.d. and analyzed with Student's *t*-test. Control: SPONGOSTAN carried with complete medium-transplantation group; EMSC: SPONGOSTAN carried with EMSCs-transplantation group.

EMSCs showed greater potentials of proliferation and differentiation than BMMSCs, and also demonstrated paracrine anti-inflammation ability by which derived various therapeutic effects of MSCs [40], [41]. To further confirm the potential of clinical application of EMSCs, we first investigated the osteogenesis potential of EMSCs using bone fracture model. EMSCs were seeded on a collagen-based gelatin sponge (SPONGOSTAN) and implanted into the fracture site. 14 days after surgery, osteocalcification of the injured sites was evaluated by X-ray image, and significantly higher bone density was observed in the EMSCs treated group indicating that EMSCs can improve bone repair response (Figure 8A, 8B). Histological examination of fracture sites revealed that newly formed bone tissue and capillaries were formed in the EMSCs implanted group while the control group showed the presence of disorganized tissue accompanied with more leukocytes infiltration (Figure 8C).

**Figure 9:**
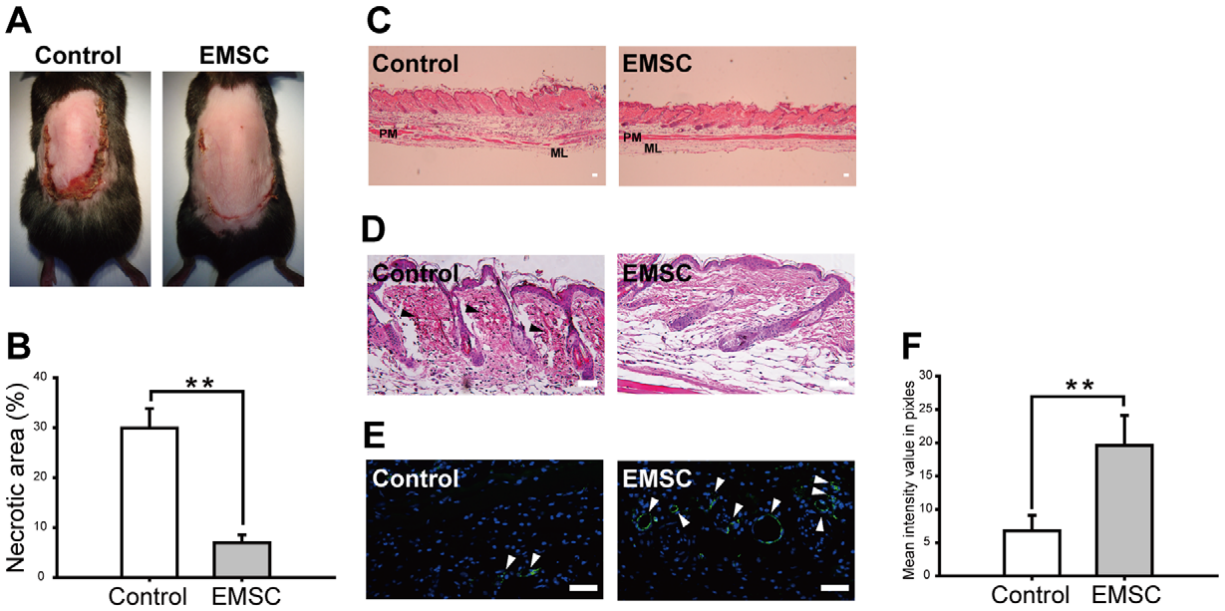
Prevention of skin flap necrosis in mice by EMSCs injection. (A) Ischemic flap showed necrosis in the control mice and near complete healing in the EMSCs (at the fifth passage) injected mice after 6 days of surgery. (B) Percentage of necrosis was quantified by planimetry. Percentage of necrotic area is significantly larger in the control group than in the EMSCs injected group (n = 4). (C) The skin structure was evaluated by H&E staining. Skin thickness is decreased and mucinous layer is preserved in the EMSCs injected group as compared to the control group. (D) In higher magnification, extra vascular erythrocytes and hemorrhage (arrowhead) can be observed in the control group. (E) Immunostaining of von Willebrand factor (vWF) of skin flap, and (F) its quantification showed significantly more vWF expressed cells (arrowhead) in the EMSCs injected group than the control group. **represents *P*<0.01. Scale bars represent 100 µm. Data are presented as mean ± s.d. and analyzed with Student's *t*-test. PM: platysma muscle; ML: mucinous layer; Control: complete medium injection-group; EMSC: EMSCs-injection group.

Recent studies showed MSCs could enhance angiogenesis and that play a pivotal role in repairing ischemic tissue damage and wound healing [42], [43]. Therefore, we used mouse dorsal skin flap to investigate the potential therapeutic effects of EMSCs in ischemic chronic wound. In consistent with the results of other sources of MSCs [44], EMSCs improved wound healing at incisions (Figure 9A) and significantly reduced necrotic area (6.95±1.6%) as compared with the control group (29.9±3.94%) (Figure 9B). Skin thickness in the EMSCs injected group was decreased and the mucinous layer was enriched below the platysma muscle when compared to the control group (Figure 9C). More severe hemorrhages and erythrocyte extravasations in the dermis could be observed in the control group than in the EMSCs injected group (Figure 9D). Immunostaining of von Willebrand factor (vWF) indicated a significantly increased capillary density in the EMSCs injected group (Figure 9E, 9F), and suggested that EMSCs have beneficial effects on chronic ischemic wound healing probably by promoting angiogenesis.

**Figure 10:**
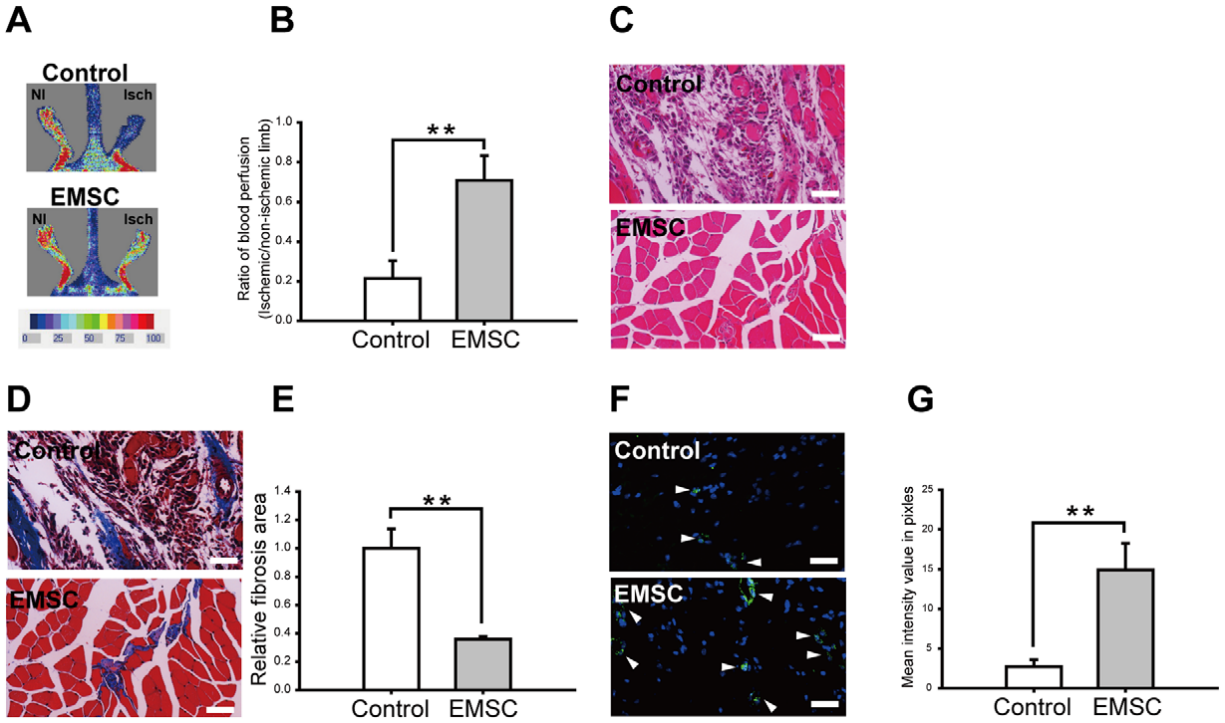
Transplanted EMSCs improved blood perfusion in ischemic limb. (A) Foot perfusion was evaluated by laser Doppler blood perfusion analysis at day 7 post ischemia. In color-coded images, red represents normal perfusion, while dark blue represents low or absent perfusion. (B) Quantification of the foot perfusion showed that EMSCs (at the fifth passage) transplantation significantly improved blood perfusion (n = 3). (C) H&E staining showed massive muscle degeneration in ischemic regions in the control group compared to the markedly reduced muscle degeneration in the EMSCs group. (D) Masson's Trichrome staining and (E) its quantification showed significantly larger fibrotic area in the control group than the EMSCs injected group. (F) Immunostaining of von Willebrand factor (vWF) of ischemic limb muscle and (G) its quantification showed significantly more vWF expressed cells (arrowhead) in the EMSCs injected group than the control group. Scale bars represent 100 µm. **represents *P*<0.01. Data are presented as mean ± s.d. and analyzed with Student's *t*-test. Isch: ischemic limb; NI: non-ischemic limb; Control: complete medium injection-group; EMSC: EMSCs-injection group.

A mouse hindlimb ischemia was also used to further confirm the effect of EMSCs on angiogenesis *in vivo*. Seven days after the onset of ischemia, laser Doppler perfusion imaging showed significant recovery of perfusion in the EMSCs injected group (0.71±0.12, relative to the non-ischemic limb) as compared to the control group (0.21±0.09) (Figure 10A, 10B). H&E staining showed muscle degeneration, and Masson's Trichrome staining showed severe fibrosis in the ischemic region in the control group. On the other hand, significantly reduced muscle degeneration and minimal fibrosis were observed in the EMSCs treated group (Figure 10C–10E). In consistent with the results from skin flap model, immunostaining of vWF of ischemic muscle indicated an increase in capillary density in the EMSCs injected group (Figure 10F, 10G), and supported that EMSCs could promote angiogenesis to improve in blood perfusion and muscle recovery in ischemic hindlimbs.

## Discussion

MSCs can serve as progenitors for bone, cartilage, adipose, muscle and other tissues [45], [46], [47]. Despite the use of BMMSCs as a common adult stem cell source, murine BM cells are more difficult to isolate and culture than those of other species due to low frequency and mixed cell contamination [48], [49], [50]. Therefore the use of BMMSCs requires *ex vivo* cell expansion, and usually results in more senescent cells. In addition to bone marrow, researchers also tried to isolate MSCs from compact bone [51] or trabecular bone [52], [53] to overcome this limitation. The purpose of this study was to find alternative murine MSCs sources that provide sufficient MSCs in earlier passages. In this study, we demonstrated that the epiphysis serves as an easy-access and rich stem cell reservoir, resolving difficulties that arise from isolating and expanding murine BMMSCs. To verify MSCs establishment, EMSCs were analysed for general MSCs features and their key capabilities were compared with those of BMMSCs.

The minimum criteria for defining MSCs includes three parameters: clonogenic capabilities, specific surface antigens identification and differentiation potentials [54]. Experimental results revealed that EMSCs exhibited significantly stronger colony- forming ability, not only in numbers but also in size, when compared with BMMSCs. In addition, proliferation capability was determined by PDT assay. We found that BMMSCs growth decreased after few passages, whereas EMSCs continued to grow rapidly. Hence, EMSCs might have an advantage over BMMSCs in providing a large quantity of cells in a short culture period.

We demonstrated through differentiation studies that EMSCs have the potentials to differentiate into adipogenic, osteogenic and chondrogenic cell lineages. EMSCs showed significantly better differentiation capabilities than BMMSCs, especially in adipogenesis and chondrogenesis. In adipogenesis, EMSCs formed lipid droplets more readily, and the number and size of lipid droplets in EMSCs were more than in BMMSCs. Accordingly, EMSCs expressed higher level of early (PPARγ) and late (LPL) adipogenesis markers than BMMSCs. In chondrogenesis, EMSCs showed deeper coloured and larger mast cell accumulation on toluidine blue staining. These results suggest that EMSCs exhibit higher differentiation potentials than BMMSCs.

We examined the expression of EMSCs markers and found that some differed from those of BMMSCs. The data confirmed that EMSCs cultured in our system gave rise to a population of cells with MSCs characteristics. In consistent with general MSCs profiles, EMSCs were positive for CD29, CD44 and CD73, and negative for CD11b, CD45 and CD31, suggesting that the established EMSCs were not from hematopoietic and endothelial lineages. EMSCs exhibited higher and more homogeneous Sca-1 expression might be related to their high proliferation capabilities [55], [56]. In accordance of previous studies, CD105 and CD166 were demonstrated to relate to chondrogenic potential [57], [58] and our data also indicated that EMSCs, with CD166 positive, were superior in chondrogenic potential. Notably, EMSCs were negative for MHC I expression under our culture condition, whereas BMMSCs were positive. When treated with IFN-γ, a cytokine known to enhance the MHC molecules expression, EMSCs were found to be positive for MHC I, implying that MHC I molecules exist in the cytoplasm, but not on the cell surface. Following changes in phenotype that occured over several passages revealed that CD73, CD166, SSEA-4 and Sca-1 decreased with increased propagation, and grew in similarity to BMMSCs while the MHC I expression was still negative for EMSCs. In conclusion, our established EMSCs can be classified as mesenchymal stem cells and were distinct from BMMSCs.

In principle, cellular aging can be accelerated with increased propagating and can affect amplified differentiation potential [59], [60]. Our results showed that cultured EMSCs showed less SA-β-gal positive cells than BMMSCs over several passages. Cyclin-dependent kinase markers, p16 and p21, were important for terminal growth arrest, and EMSCs showed lower p16 and p21 expression than BMMSCs regardless of primary or longer passages.

Due to the potentials of multilineage differentiation and low immunogenicity, MSCs draw attention for the use in regenerative medicine. Recent researches also demonstrated that some therapeutic effects of MSCs may derive from their paracrine effects [61], [62]. In our study, EMSCs not only showed great proliferation and differentiation potentials but also demonstrated immunosuppressive and anti-inflammation capacities through a paracrine effect. Thus, it is reasonable to expect that EMSCs are at least as potential as other MSCs for cell therapy. We evaluated the therapeutic effects of EMSCs in three animal models, including bone fracture, skin flap and hindlimb ischemia models. Our study showed that transplanted EMSCs accelerated the fracture healing process and enhanced osteocalcification indicating that EMSCs can promote fracture repair. It has been shown that MSCs contribute to angiogenesis directly by participating in new vessel formation, or indirectly by secreting angiogenic factors [63], [64], [65]. Therefore, we used dorsal skin flap model, in which an oxygen tension gradient occurs in the ischemic tissue [66], to investigate the effects of EMSCs on the neovascularization *in vivo*. Transplanted EMSCs obviously reduced necrosis and almost fully protected the edge of the flap. In addition, EMSCs effectively preserved skin structures including the mucinous layer which is important for skin water content and tissue structural integrity [67]. In hindlimb ischemia model, EMSCs improved blood perfusion and reduced muscle degeneration and tissue fibrosis. Moreover, EMSCs treatment significantly increased vWF positive capillary cells in both skin flap and hindlimb ischemia models, suggesting that EMSCs enhance neovascularization and might play a beneficial role in ischemic/hypoxia environment.

Collectively, EMSCs were characterized as MSCs due to their capacities for differentiation into multiple lineages and cell surface marker expression profiles, while EMSCs were found to have greater abilities to proliferate and differentiate than BMMSCs. Our data demonstrates that EMSCs provide an efficient and reproducible method for obtaining MSCs with high proliferating potential, and hence EMSCs can be used as an alternative to MSCs for cell therapy. We provide a novel and reliable method for preparing an improved population of murine MSCs.

## Materials and Methods

### Animals and Ethics Statement

C57BL/6 and BALB/c mice of 6–8 weeks were purchased from the Laboratory Animal Center of Medical College in National Taiwan University (Taipei, Taiwan). Mice were kept under standard conditions, and all experimental procedures were approved by the Institutional Animal Care and Use Committee (IACUC) of National Taiwan University (approval number 96-EL-22-NTU).

### Isolation and Culture of EMSCs

C57BL/6 mice were sacrificed by cervical dislocation. The femurs and tibias were isolated and the adherent soft tissues on femurs and tibias were removed thoroughly. The epiphyses were then removed by a shears and transferred to 22.1 cm^2^ culture dish (TPP, Trasadingen, Switzerland) and cultured in complete medium composed of MEM alpha (Sigma-Aldrich, St. Louis, MO) supplemented with 20% fetal bovine serum (FBS; ECS tested; Hyclone, Logan, UT), 2 mM L-glutamine (Invitrogen, Carlsbad, CA), 100 U/ml penicillin, and 100 µg/ml streptomycin (Invitrogen). The cells were incubated in a humidified atmosphere containing 95% air and 5% CO_2_ at 37°C. The non-adherent cells were removed by changing the medium every 3 days. After 11 days, the primary culture reached approximately 70% confluence, and the cells were detached by 0.25% trypsin/0.1 mM ethylenediaminetetraacetic acid (trypsin/EDTA; Invitrogen) for 1 min at 37°C. After that, the reaction was stopped by adding complete medium. The cells that cannot be lifted within 1 min were discarded. The detached cells were centrifuged at 400 *g* for 5 min and resuspended in the complete medium and were ready for the transient lower-density plastic adherence (tLDA) purification as described below.

### Isolation and Culture of BMMSCs

BMMSCs were obtained as previously described [39], [68]. In brief, the femurs and tibias were isolated from cervical dislocated C57BL/6 mice and the adherent soft tissues were cleaned thoroughly. Then the epiphyses were removed by a rongeur. The marrow cells were harvested from diaphysis by inserting a syringe needle (23-gauge and 26-gauge for femurs and tibias, respectively) and flushing with complete medium consisting of MEM alpha (Sigma-Aldrich) supplemented with 20% FBS (Hyclone), 2 mM L-glutamine (Invitrogen), 100 U/ml penicillin, and 100 µg/ml streptomycin (Invitrogen). We plated the cells onto 60 cm^2^ culture dishes (TPP) at a density of 2×10^5^ cells/cm^2^. The cells were incubated in a humidified atmosphere containing 95% air and 5% CO_2_ at 37°C. The non-adherent cells were removed by changing medium every 3 days. When these primary cultures reached 70% confluence, the cells were lifted by incubating with 0.25% trypsin/EDTA (Invitrogen) for 1 min at 37°C. These cells were ready for tLDA purification as described below.

### tLDA Approach

The tLDA approach has been described previously [39]. Briefly, the lifted cells from the primary culture were passed through a 40 µm mesh (BD Biosciences, San Jose, CA) and then the filtered single cells were replated onto 60 cm^2^ culture dishes at a density of 1.25×10^4^ cells/cm^2^. These cells were allowed to adhere for 2–3 h at 37°C in a humidified atmosphere containing 95% air and 5% CO_2_. Afterwards, the cells were lifted by incubating with 0.025% trypsin/EDTA (Invitrogen) for 5 min at 37°C and were seeded at a density of 5×10^4^ cells/cm^2^ for subsequent analysis.

### Population Doubling Time (PDT)

The mean PDT was calculated according to the equation: TD = *t*_p_log2/(logNt−logNo), where Nt is the number of cells harvested, No is the number of cells inoculated, and t is the time of the culture (in hour). The trypan blue exclusion test was used to count the cells with the hemocytometer [69].

### Colony Formation Assay

For determination of colony forming units (CFUs), cells were plated at a density of 150 cells/9.01 cm^2^ culture dish (TPP). After incubation for 9 days, the colonies formed were fixed by methanol (Sigma-Aldrich) and stained with Geimsa solution (Sigma-Aldrich). A cluster of at least 20 cells was defined as a CFU. CFU sizes and CFU number were enumerated by a light microscope. To determine the relationship between CFU size and cell number, CFU was categorized depending on cell number per CFU: 20–50 cells/CFU, 51–100 cells/CFU, 101–150 cells/CFU and >151 cells/CFU.

### *In Vitro* Differentiation

To induce adipogenic differentiation, cells were cultured to near confluence and cultured in adipogenic induction medium consisting of MEM alpha (Sigma-Aldrich) supplemented with 10% FBS (Hyclone), 10 µg/ml insulin (Sigma-Aldrich), 1 µM dexamethasone (Sigma-Aldrich), 0.5 mM isobutyl-methylxanthine (Sigma-Aldrich), and 100 µM indomethacin (Sigma-Aldrich) for 7 days. The induction medium was changed every 3 days [29]. At the end of the differentiation period, cells were fixed with 10% formalin for 10 min and lipid droplets were stained by Oil Red O (Sigma-Aldrich) staining. To quantify the Oil Red O staining results, we extracted the Oil Red O from lipid droplets by DMSO (Sigma-Aldrich) and measured the absorbance by SpectraMax 190 ELISA plate reader (Molecular Devices, Sunnyvale, CA) at 550 nm.

To induce osteogenic differentiation, the confluent cells were incubated in osteogenic induction medium consisting of MEM alpha (Sigma-Aldrich) supplemented with 10% FBS (Hyclone), 0.1 µM dexamethasone (Sigma-Aldrich), 10 mM β-glycerolphosphate (Sigma-Aldrich) and 50 µM ascorbic acid (Sigma-Aldrich) for 7 days [29]. The induction medium was changed every 3 days. The bone matrix mineralization was evaluated by Alizarin red S (ARS; Sigma-Aldrich) staining. The ARS was extracted by adding 10% cetylpyridinium chloride (Sigma) in 8 mM Na_2_HPO_4_ (Merck, Darmstadt, Germany) and 1.5 mM KH_2_PO_4_ (Merck) and the absorbance was measured by SpectraMax 190 ELISA plate reader (Molecular Devices) at 550 nm.

To induce chondrogenic differentiation, a pellet culture system was used to differentiate. Chondrogenic induction medium consisted of MEM alpha supplemented with 1% FBS (Hyclone), 6.25 µg/ml insulin (Sigma-Aldrich), 50 µM ascorbic acid (Sigma-Aldrich), and 10 ng/ml TGF-β1 (R&D Systems, Minneapolis, MN). The induction medium was changed twice per week. The production of proteoglycan was characterized by toluidine blue (Sigma-Aldrich) staining [70].

### Flow Cytometric Analysis

Cells were stained with fluorescent isothiocyanate (FITC) or PE-conjugate monoclonal antibodies (Table 1) for 30 min at 4°C in dark according to the product instructions. Ten thousand events were acquired on a FACSCalibur (BD Biosciences, Franklin Lakes, NJ), and analyzed by FCS Express software (Version 4.0; Denovo software, Los Angeles, CA). All experiments included negative controls without antibodies or with isotype controls (eBioscience, San Diego, CA).

**Table 1:** List of Antibodies Used in Flow Cytometry Analysis.

Antibody	Clone	Ref. No.	Conjugated	Isotype	Supplier
CD11b	M1/70	12-0112	PE	Rat IgG2b	eBioscience
CD29	HMb1-1	12-0291	PE	Armenian Hamster IgG	eBioscience
CD31	390	12-0311	PE	Rat IgG2a	eBioscience
CD34	RAM34	560238	FITC	RatIgG2a	BD Pharmingen
CD44	IM7	12-0441	PE	Rat IgG2b	eBioscience
CD45	30-F11	12-0451	PE	Rat IgG2b	eBioscience
CD73	TY/11.8	12-0731	PE	Rat IgG1	eBioscience
CD105	MJ7/18	12-1051	PE	Rat IgG2a	eBioscience
CD106	429	11-1061	FITC	Rat IgG2a	eBioscience
CD117	2B8	12-1171	PE	Rat IgG2b	eBioscience
CD166	eBioALC-48	12-1661	PE	Rat IgG2a	eBioscience
Sca-1	D7	12-5981	PE	Rat IgG2a	eBioscience
SSEA-4	MC-813-70	12-8843	PE	Mouse IgG3	eBioscience
MHC class I	34-1-2S	12-5998	PE	Mouse IgG2a	eBioscience
MHC class II	M5/114.15.2	12-5321	PE	Rat IgG2b	eBioscience

### Quantitative Real Time Reverse Transcription-Polymerase Chain Reaction (qPCR)

Total cellular RNA was extracted using TRIzol reagent (Invitrogen) and then treated with RNase free DNase Set (Promega, Madison, MI) according to manufacturer's instructions. Reverse transcription reactions were performed with 5 µg total RNA using the SuperScript First-Strand Synthesis System (Invitrogen), according to the manufacturer's instructions. Real-time PCR (ABI PRISM 7000; Applied Biosystems; Foster City, CA) was performed with 1 µL of the single-stranded cDNA sample with SYBR Green PCR master mix (Applied Biosystems). The sequences of primers used were as follows: forward 5′-CATGGCCTTCCGTGTTCCTA-3′, reverse 5′-GCGGCACGTCAGATCCA-3′ for *Glyceraldehyde-3-phosphate dehydrogenase* (*GAPDH*); forward 5′-ACATAAAGTCCTTCCCGCTGACCA-3′, reverse 5′-AAATTCGGATGGCCACCTCTTTGC-3′ for *peroxisome proliferator activated receptor gamma* (*PPARγ*); forward 5′-ACGAGCGCTCCATTCATCTCTTCA-3′, reverse 5′-TCTTGCTGCTTCTCTTGGCTCTGA-3′ for *lipoprotein lipase* (*LPL*); forward 5′-GCTTTCATTAGGCAGGGCCAACAA-3′, reverse 5′- AGGGCTGGATCTCAAACTCACACA-3′ for *runt related transcription factor 2* (*Runx2*); forward 5′-TTCTCCTGGCAAAGACGGACTCAA-3′, reverse 5′- AGGAAGCTGAAGTCATAACCGCCA-3′ for *collagen, type I, alpha 1* (*Col1a1*); forward 5′-GAACTCTTTCGGTCGTACCC-3′, reverse 5′-CGAATCTGCACCGTAGTTGA-3′ for *p16*; forward 5′- CCAGGCCAAGATGGTGTCTT-3′, reverse 5′-TGAGAAAGGATCAGCCATTGC-3′ for *p21*. Annealing temperature was 60°C. Each amplification reaction was checked to confirm the absence of nonspecific PCR product by melting curve analysis. The relative gene expression level was calculated and presented with the 2^−ΔΔCt^ method. *GAPDH* was used as a reference gene to normalize specific gene expression in each sample.

### Quantification of Telomere Length

Average telomere length (ATL) was measured by real-time PCR from total genomic mouse DNA as previous study [71]. In brief, total genomic DNA was extracted using Dneasy Blood and Tissue Kit (Qiagen, Hilden, Germany) according to manufacturer's instructions. 20 ng samples of each DNA were performed by real-time PCR (ABI PRISM 7000; Applied Biosystems) with SYBR Green PCR master mix (Applied Biosystems). The average telomere length ratio was determined by the level of telomeric DNA normalized to the quantity of a single copy gene, the *acidic ribosomal phosphoprotein PO* (*36B4*). Forward and reverse telomeric primers were 5′-CGGTTTGTTTGGGTTTGGGTTTGGGTTTGGGTTTGGGTT-3′ and 5′-GGCTTGCCTTACCCTTACCCTTACCCTTACCCTTACCCT-3′ respectively. Forward and reverse primers of *36B4* gene were 5′-ACTGGTCTAGGACCCGAGAAG-3′ and 5′-TCAATGGTGCCTCTGGAGATT -3′, respectively.

### Telomerase Activity

Telomerase activity was performed in triplicates with the TRAPeze® XL Telomerase Detection kit (Millipore, Billerica, MA) as the manufacturer's instructions. Briefly, cells were lysed by CHAPS lysis buffer and incubated on ice for 30 min. Then, cells were centrifuged at 12,000 *g* at 4°C, and the supernatant was assayed for telomerase. For PCR amplification, each reaction volume is 50 µl, containing 10 µl of the 5× TRAPeze® XL Reaction Mix, 0.4 µl of Tag Polymerase (5 U/ml; Invitrogen), 37.6 µl of ddH_2_O, and 2 µl of the sample extracted. In a themocycler (ABI 2700; Applied Biosystems), performed a 4-step PCR at 94°C for 30 s, 59°C for 30 s, 72°C for 1 min for 36 cycles, followed by a 72°C for 3 min extension step and then 55°C for 25 min, concluding with a 4°C incubation. The fluorescence of each reaction was detected with a Fluoroskan Ascent FL fluorescent plate reader (Labsystems; Farnborough, UK). The telomerase activity was determined by calculating the log of the relative ratio net fluorescence increase (Δ fluorescein absorbance/Δ sulforhodamine absorbance) for each reaction described detail in the manufacturer's instructions

### SA-β-Gal Staining

Cells were grown to 50% confluence and stained by senescence cells histochemical staining kit (Sigma-Aldrich) as manual described. Briefly, cells were fixed in fixation buffer, washed with PBS and stained the cells by incubating in freshly prepared staining mixture at 37°C without O_2_ overnight.

### Mitogen Proliferation Assays and Allogeneic Mixed Lymphocyte Reaction (MLR)

The splenocytes from C57BL/6 and BALB/c mice were prepared using Ficoll-Paque density centrifugation (1.077 g/ml; Amersham Pharmacia Biotech, NJ) followed by two washes in RPMI 1640 (Invitrogen) supplemented with 50 µM 2-mercaptoethanol (Sigma-Aldrich), 10% FBS (Hyclone), 100 U/ml penicillin, and 100 µg/ml streptomycin (Invitrogen). A graded number of mitomycin C (Sigma-Aldrich)-treated EMSCs or BMMSCs were seeded in triplicate in flat-bottom 96-well plates and maintained at 37°C for 6 h. In mitogen proliferation assays, splenocytes (2×10^5^ cells/well) from C57BL/6 or BALB/c mice treated with 5 µg/ml Con A (Sigma-Aldrich) were cultured with or without EMSCs or BMMSCs from C57BL/6 mice. In MLR, responding splenocytes (2×10^5^ cells/well) from C57BL/6 mice and an equal number of mitomycin C-treated allogeneic stimulating splenocytes from BALB/c mice were added into the EMSCs or BMMSCs culture. Proliferation assays were performed after 4 or 5 days using the CellTiter 96 Aqueous Nonradioactive Cell Proliferation Assay kit (Promega, Madison, WI), according to the manufacturer's instructions. The absorbance was measured by SpectraMax 190 ELISA plate reader (Molecular Devices) at 490 nm.

### Transwell and Coculture Assay

EMSCs (2×10^4^ cells/ml) were cultured in an upper chamber of the Transwell insert (Millipore) with or without splenocytes (2×10^6^ cells/ml) cultured in a lower chamber. The transwell membrane was semipermeable with a pore size of 0.4 µm. After three days of culture, splenocytes were collected for mRNA analysis.

### Bone Fracture Model

Eight-week-old female C57BL/6 mice were subjected to a bone fracture model as previously described [72], [73]. Briefly, a closed transverse fracture was performed in the midsection of the femur by a motorized drill (2 mm in diameter) and fractured femurs were reconnected by a pin (25-gauge needle). EMSCs (5×10^5^ cells/mouse) were plated on 2 mm×2 mm SPONGOSTAN (Johnson & Johnson, New Jersey, USA) at 37°C for 2 h and then transplanted by covering on the fracture site. Experimented mice were examined by X-ray (SkyScan 1076; SkyScan, Belgium) 14 days post-transplantation and the bone density of the fracture site was assessed using ImageJ software (NIH). Femurs were collected for hematoxylin and eosin (H&E) staining.

### Skin Flap Model

Eight-week-old female C57BL/6 mice underwent skin flap surgery at their back as previously described [74], [75]. In brief, a peninsular-shaped flap (3 cm×2 cm) was elevated on mouse dorsal skin and two vascular pedicles from the lateral thoracic arteries were sectioned. Following surgery, the flap was injected with 200 µl of EMSCs (5×10^5^ cells/mouse) or complete medium intradermally using 29-guage needle. The injected solution was separated at four sites near the vascular cutting sites. Mice were photographed for six days after the surgery and then sacrificed to collect flap tissues for H&E staining and immunostaining. The necrotic area was quantified using the ImageJ software (NIH).

### Hindlimb Ischemia Model

Eight-week-old female C57BL/6 mice were subjected to surgical ligature at the proximal end of the left femoral artery as previously described [76], [77]. Seven days after surgery, 100 µl of EMSCs (2×10^6^ cells/mouse) or complete medium was injected intramuscularly into 5 sites of the gracilis muscle in the medial thigh immediately. Laser Doppler perfusion imaging (moorLDI2; Moor Instruments Ltd., Devon, UK) was performed seven days after the surgery and ischemic limb muscles were then harvested for immunohistochemistry analysis. H&E staining and Masson's Trichrome (Sigma-Aldrich) staining were performed, and fibrosis area was calculated with ImageJ software (NIH).

### Immunohistochemical Staining

Samples were embedded in paraffin blocks. Tissue slides were deparaffinized, treated with epitope retrieval buffer (Thermal scientific, Inc., Odessa, TX) at 95°C for 25 min, and then incubated with a primary antibody, rabbit anti-von Willebrand factor (vWF; 1∶400; Abcam, Cambridge, UK), at 4°C overnight. Goat anti-rabbit conjugated Alexa-488 antibody (1∶200; Invitrogen) was used as the secondary antibody. For nuclear staining, sections were incubated in 0.1 µg/ml 4,6 diamidino-2-phenylindole (DAPI; Invitrogen) for 3 min. Fluorescence was observed microscopically and fluorescence intensity of immunostaining was evaluated with ImageJ software (NIH).

### Statistical Analysis

All experiments have more than 3 biological repeats and all values were presented as mean ± standard deviation. Statistical comparisons were analyzed with the Student's *t*-test and a *p*-value less than 0.05 was considered statistically significant.

## References

[1] Friedenstein AJ, Chailakhjan RK, Lalykina KS (1970). The development of fibroblast colonies in monolayer cultures of guinea-pig bone marrow and spleen cells.. Cell Tissue Kinet.

[2] Bianco P, Robey PG, Simmons PJ (2008). Mesenchymal stem cells: revisiting history, concepts, and assays.. Cell Stem Cell.

[3] Chamberlain G, Fox J, Ashton B, Middleton J (2007). Concise review: mesenchymal stem cells: their phenotype, differentiation capacity, immunological features, and potential for homing.. Stem Cells.

[4] Pittenger MF, Mackay AM, Beck SC, Jaiswal RK, Douglas R (1999). Multilineage potential of adult human mesenchymal stem cells.. Science.

[5] Lee KD, Kuo TK, Whang-Peng J, Chung YF, Lin CT (2004). In vitro hepatic differentiation of human mesenchymal stem cells.. Hepatology.

[6] Talens-Visconti R, Bonora A, Jover R, Mirabet V, Carbonell F (2006). Hepatogenic differentiation of human mesenchymal stem cells from adipose tissue in comparison with bone marrow mesenchymal stem cells.. World J Gastroenterol.

[7] Hisanaga E, Park KY, Yamada S, Hashimoto H, Takeuchi T (2008). A simple method to induce differentiation of murine bone marrow mesenchymal cells to insulin-producing cells using conophylline and betacellulin-delta4.. Endocr J.

[8] Chao KC, Chao KF, Fu YS, Liu SH (2008). Islet-like clusters derived from mesenchymal stem cells in Wharton's Jelly of the human umbilical cord for transplantation to control type 1 diabetes.. PLoS One.

[9] Zanini C, Bruno S, Mandili G, Baci D, Cerutti F (2011). Differentiation of mesenchymal stem cells derived from pancreatic islets and bone marrow into islet-like cell phenotype.. PLoS One.

[10] Lu P, Blesch A, Tuszynski MH (2004). Induction of bone marrow stromal cells to neurons: differentiation, transdifferentiation, or artifact?. J Neurosci Res.

[11] Greco SJ, Zhou C, Ye JH, Rameshwar P (2007). An interdisciplinary approach and characterization of neuronal cells transdifferentiated from human mesenchymal stem cells.. Stem Cells Dev.

[12] Nauta AJ, Fibbe WE (2007). Immunomodulatory properties of mesenchymal stromal cells.. Blood.

[13] Castillo MD, Trzaska KA, Greco SJ, Ponzio NM, Rameshwar P (2008). Immunostimulatory effects of mesenchymal stem cell-derived neurons: implications for stem cell therapy in allogeneic transplantations.. Clin Transl Sci.

[14] Patel SA, Sherman L, Munoz J, Rameshwar P (2008). Immunological properties of mesenchymal stem cells and clinical implications.. Arch Immunol Ther Exp (Warsz).

[15] Zhao S, Wehner R, Bornhauser M, Wassmuth R, Bachmann M (2010). Immunomodulatory properties of mesenchymal stromal cells and their therapeutic consequences for immune-mediated disorders.. Stem Cells Dev.

[16] Uccelli A, Laroni A, Freedman MS (2011). Mesenchymal stem cells for the treatment of multiple sclerosis and other neurological diseases.. Lancet Neurol.

[17] Ben-Ami E, Berrih-Aknin S, Miller A (2011). Mesenchymal stem cells as an immunomodulatory therapeutic strategy for autoimmune diseases.. Autoimmun Rev.

[18] Williams JT, Southerland SS, Souza J, Calcutt AF, Cartledge RG (1999). Cells isolated from adult human skeletal muscle capable of differentiating into multiple mesodermal phenotypes.. Am Surg.

[19] Erices A, Conget P, Minguell JJ (2000). Mesenchymal progenitor cells in human umbilical cord blood.. Br J Haematol.

[20] Peters R, Wolf MJ, van den Broek M, Nuvolone M, Dannenmann S (2010). Efficient generation of multipotent mesenchymal stem cells from umbilical cord blood in stroma-free liquid culture.. PLoS One.

[21] Gronthos S, Mankani M, Brahim J, Robey PG, Shi S (2000). Postnatal human dental pulp stem cells (DPSCs) in vitro and in vivo.. Proc Natl Acad Sci U S A.

[22] Zuk PA, Zhu M, Mizuno H, Huang J, Futrell JW (2001). Multilineage cells from human adipose tissue: implications for cell-based therapies.. Tissue Eng.

[23] Lopatina T, Kalinina N, Karagyaur M, Stambolsky D, Rubina K (2011). Adipose-derived stem cells stimulate regeneration of peripheral nerves: BDNF secreted by these cells promotes nerve healing and axon growth de novo.. PLoS One.

[24] In 't Anker PS, Scherjon SA, Kleijburg-van der Keur C, Noort WA, Claas FH (2003). Amniotic fluid as a novel source of mesenchymal stem cells for therapeutic transplantation.. Blood.

[25] Moorefield EC, McKee EE, Solchaga L, Orlando G, Yoo JJ (2011). Cloned, CD117 Selected Human Amniotic Fluid Stem Cells Are Capable of Modulating the Immune Response.. PLoS One.

[26] Yoshimura H, Muneta T, Nimura A, Yokoyama A, Koga H (2007). Comparison of rat mesenchymal stem cells derived from bone marrow, synovium, periosteum, adipose tissue, and muscle.. Cell Tissue Res.

[27] Lapi S, Nocchi F, Lamanna R, Passeri S, Iorio M (2008). Different media and supplements modulate the clonogenic and expansion properties of rabbit bone marrow mesenchymal stem cells.. BMC Res Notes.

[28] Zeng L, Rahrmann E, Hu Q, Lund T, Sandquist L (2006). Multipotent adult progenitor cells from swine bone marrow.. Stem Cells.

[29] Peister A, Mellad JA, Larson BL, Hall BM, Gibson LF (2004). Adult stem cells from bone marrow (MSCs) isolated from different strains of inbred mice vary in surface epitopes, rates of proliferation, and differentiation potential.. Blood.

[30] Sun S, Guo Z, Xiao X, Liu B, Liu X (2003). Isolation of mouse marrow mesenchymal progenitors by a novel and reliable method.. Stem Cells.

[31] Phinney DG (2008). Isolation of mesenchymal stem cells from murine bone marrow by immunodepletion.. Methods Mol Biol.

[32] Nakamura Y, Arai F, Iwasaki H, Hosokawa K, Kobayashi I (2010). Isolation and characterization of endosteal niche cell populations that regulate hematopoietic stem cells.. Blood.

[33] Kitano Y, Radu A, Shaaban A, Flake AW (2000). Selection, enrichment, and culture expansion of murine mesenchymal progenitor cells by retroviral transduction of cycling adherent bone marrow cells.. Exp Hematol.

[34] Baxter MA, Wynn RF, Jowitt SN, Wraith JE, Fairbairn LJ (2004). Study of telomere length reveals rapid aging of human marrow stromal cells following in vitro expansion.. Stem Cells.

[35] Digirolamo CM, Stokes D, Colter D, Phinney DG, Class R (1999). Propagation and senescence of human marrow stromal cells in culture: a simple colony-forming assay identifies samples with the greatest potential to propagate and differentiate.. Br J Haematol.

[36] Ornitz DM, Marie PJ (2002). FGF signaling pathways in endochondral and intramembranous bone development and human genetic disease.. Genes Dev.

[37] Olsen BR, Reginato AM, Wang W (2000). Bone development.. Annu Rev Cell Dev Biol.

[38] Shapiro F (2008). Bone development and its relation to fracture repair. The role of mesenchymal osteoblasts and surface osteoblasts.. Eur Cell Mater.

[39] Hsiao FS, Cheng CC, Peng SY, Huang HY, Lian WS (2010). Isolation of therapeutically functional mouse bone marrow mesenchymal stem cells within 3 h by an effective single-step plastic-adherent method.. Cell Prolif.

[40] da Silva Meirelles L, Caplan AI, Nardi NB (2008). In search of the in vivo identity of mesenchymal stem cells.. Stem Cells.

[41] Caplan AI (2007). Adult mesenchymal stem cells for tissue engineering versus regenerative medicine.. J Cell Physiol.

[42] Wu Y, Chen L, Scott PG, Tredget EE (2007). Mesenchymal stem cells enhance wound healing through differentiation and angiogenesis.. Stem Cells.

[43] Chen L, Tredget EE, Wu PY, Wu Y (2008). Paracrine factors of mesenchymal stem cells recruit macrophages and endothelial lineage cells and enhance wound healing.. PLoS One.

[44] Uysal AC, Mizuno H, Tobita M, Ogawa R, Hyakusoku H (2009). The effect of adipose-derived stem cells on ischemia-reperfusion injury: immunohistochemical and ultrastructural evaluation.. Plast Reconstr Surg.

[45] Bianco P, Gehron Robey P (2000). Marrow stromal stem cells.. J Clin Invest.

[46] Gronthos S, Simmons PJ (1996). The biology and application of human bone marrow stromal cell precursors.. J Hematother.

[47] Prockop DJ (1997). Marrow stromal cells as stem cells for nonhematopoietic tissues.. Science.

[48] Meirelles Lda S, Nardi NB (2003). Murine marrow-derived mesenchymal stem cell: isolation, in vitro expansion, and characterization.. Br J Haematol.

[49] Phinney DG, Kopen G, Isaacson RL, Prockop DJ (1999). Plastic adherent stromal cells from the bone marrow of commonly used strains of inbred mice: variations in yield, growth, and differentiation.. J Cell Biochem.

[50] Eslaminejad MB, Nikmahzar A, Taghiyar L, Nadri S, Massumi M (2006). Murine mesenchymal stem cells isolated by low density primary culture system.. Dev Growth Differ.

[51] Zhu H, Guo ZK, Jiang XX, Li H, Wang XY (2010). A protocol for isolation and culture of mesenchymal stem cells from mouse compact bone.. Nat Protoc.

[52] Sakaguchi Y, Sekiya I, Yagishita K, Ichinose S, Shinomiya K (2004). Suspended cells from trabecular bone by collagenase digestion become virtually identical to mesenchymal stem cells obtained from marrow aspirates.. Blood.

[53] Tuli R, Tuli S, Nandi S, Wang ML, Alexander PG (2003). Characterization of multipotential mesenchymal progenitor cells derived from human trabecular bone.. Stem Cells.

[54] Horwitz EM, Le Blanc K, Dominici M, Mueller I, Slaper-Cortenbach I (2005). Clarification of the nomenclature for MSC: The International Society for Cellular Therapy position statement.. Cytotherapy.

[55] Epting CL, King FW, Pedersen A, Zaman J, Ritner C (2008). Stem cell antigen-1 localizes to lipid microdomains and associates with insulin degrading enzyme in skeletal myoblasts.. J Cell Physiol.

[56] Holmes C, Stanford WL (2007). Concise review: stem cell antigen-1: expression, function, and enigma.. Stem Cells.

[57] Ozbey O, Sahin Z, Acar N, Ustunel I (2010). Distribution of CD105 and CD166 positive cells in the proximal epiphysis of developing rat humerus.. Histol Histopathol.

[58] Alsalameh S, Amin R, Gemba T, Lotz M (2004). Identification of mesenchymal progenitor cells in normal and osteoarthritic human articular cartilage.. Arthritis Rheum.

[59] Li Z, Liu C, Xie Z, Song P, Zhao RC (2011). Epigenetic dysregulation in mesenchymal stem cell aging and spontaneous differentiation.. PLoS One.

[60] Wilson A, Shehadeh LA, Yu H, Webster KA (2010). Age-related molecular genetic changes of murine bone marrow mesenchymal stem cells.. BMC Genomics.

[61] Granero-Molto F, Myers TJ, Weis JA, Longobardi L, Li T (2011). Mesenchymal stem cells expressing insulin-like growth factor-I (MSCIGF) promote fracture healing and restore new bone formation in Irs1 knockout mice: analyses of MSCIGF autocrine and paracrine regenerative effects.. Stem Cells.

[62] Granero-Molto F, Weis JA, Miga MI, Landis B, Myers TJ (2009). Regenerative effects of transplanted mesenchymal stem cells in fracture healing.. Stem Cells.

[63] Cho SW, Moon SH, Lee SH, Kang SW, Kim J (2007). Improvement of postnatal neovascularization by human embryonic stem cell derived endothelial-like cell transplantation in a mouse model of hindlimb ischemia.. Circulation.

[64] Kinnaird T, Stabile E, Burnett MS, Lee CW, Barr S (2004). Marrow-derived stromal cells express genes encoding a broad spectrum of arteriogenic cytokines and promote in vitro and in vivo arteriogenesis through paracrine mechanisms.. Circ Res.

[65] Lian WS, Cheng WT, Cheng CC, Hsiao FS, Chen JJ (2011). In vivo therapy of myocardial infarction with mesenchymal stem cells modified with prostaglandin I synthase gene improves cardiac performance in mice.. Life Sci.

[66] Ceradini DJ, Kulkarni AR, Callaghan MJ, Tepper OM, Bastidas N (2004). Progenitor cell trafficking is regulated by hypoxic gradients through HIF-1 induction of SDF-1.. Nat Med.

[67] Uzuka M, Nakajima K, Ohta S, Mori Y (1981). Induction of hyaluronic acid synthetase by estrogen in the mouse skin.. Biochim Biophys Acta.

[68] Krampera M, Glennie S, Dyson J, Scott D, Laylor R (2003). Bone marrow mesenchymal stem cells inhibit the response of naive and memory antigen-specific T cells to their cognate peptide.. Blood.

[69] Nadri S, Soleimani M, Hosseni RH, Massumi M, Atashi A (2007). An efficient method for isolation of murine bone marrow mesenchymal stem cells.. Int J Dev Biol.

[70] Johnstone B, Hering TM, Caplan AI, Goldberg VM, Yoo JU (1998). In vitro chondrogenesis of bone marrow-derived mesenchymal progenitor cells.. Exp Cell Res.

[71] Callicott RJ, Womack JE (2006). Real-time PCR assay for measurement of mouse telomeres.. Comp Med.

[72] Nagano M, Kimura K, Yamashita T, Ohneda K, Nozawa D (2010). Hypoxia responsive mesenchymal stem cells derived from human umbilical cord blood are effective for bone repair.. Stem Cells Dev.

[73] Taguchi K, Ogawa R, Migita M, Hanawa H, Ito H (2005). The role of bone marrow-derived cells in bone fracture repair in a green fluorescent protein chimeric mouse model.. Biochem Biophys Res Commun.

[74] Nagano M, Yamashita T, Hamada H, Ohneda K, Kimura K (2007). Identification of functional endothelial progenitor cells suitable for the treatment of ischemic tissue using human umbilical cord blood.. Blood.

[75] Toutain CE, Brouchet L, Raymond-Letron I, Vicendo P, Berges H (2009). Prevention of skin flap necrosis by estradiol involves reperfusion of a protected vascular network.. Circ Res.

[76] Zhang Y, Zhang R, Li Y, He G, Zhang D (2012). Simvastatin augments the efficacy of therapeutic angiogenesis induced by bone marrow-derived mesenchymal stem cells in a murine model of hindlimb ischemia.. Mol Biol Rep.

[77] Dar A, Domev H, Ben-Yosef O, Tzukerman M, Zeevi-Levin N (2012). Multipotent vasculogenic pericytes from human pluripotent stem cells promote recovery of murine ischemic limb.. Circulation.

